# Molecular Profiling of Spermatozoa Reveals Correlations between Morphology and Gene Expression: A Novel Biomarker Panel for Male Infertility

**DOI:** 10.1155/2021/1434546

**Published:** 2021-09-18

**Authors:** Nino Guy Cassuto, David Piquemal, Florence Boitrelle, Lionel Larue, Nathalie Lédée, Ghada Hatem, Léa Ruoso, Dominique Bouret, Jean-Pierre Siffroi, Alexandre Rouen, Said Assou

**Affiliations:** ^1^ART Unit, Drouot Laboratory, Paris, France; ^2^ACOBIOM, University of Montpellier, Montpellier, France; ^3^Service de Bbiologie de la Rreproduction-Préservation de la fertilitéFertilité—Andrologie, Hôpital de Poissy Saint Germain en Laye, Poissy, France; ^4^Université Paris-Saclay, UVSQ, INRAE, BREED, Jouy-en-Josas. École Nationale Vétérinaire d'Alfort, BREED, Maisons-Alfort, France; ^5^IVF Center Diaconesses-Drouot, Diaconesses Saint Simon Hospital, Paris, France; ^6^IVF Center Bluets-Drouot, Les Bluets Hospital, Paris, France; ^7^IVF Center Delafontaine-Drouot, Delafontaine Hospital, Saint-Denis, France; ^8^Département de Génétique Médicale, Sorbonne Université, INSERM, Maladies Génétiques d'Expression Pédiatrique, APHP, Hôpital d'Enfants Armand Trousseau, Paris, France; ^9^IRMB, Univ Montpellier, INSERM, CHU Montpellier, Montpellier, France

## Abstract

Choosing spermatozoa with an optimum fertilizing potential is one of the major challenges in assisted reproductive technologies (ART). This selection is mainly based on semen parameters, but the addition of molecular approaches could allow a more functional evaluation. To this aim, we used sixteen fresh sperm samples from patients undergoing ART for male infertility and classified them in the high- and poor-quality groups, on the basis of their morphology at high magnification. Then, using a DNA sequencing method, we analyzed the spermatozoa methylome to identify genes that were differentially methylated. By Gene Ontology and protein–protein interaction network analyses, we defined candidate genes mainly implicated in cell motility, calcium reabsorption, and signaling pathways as well as transmembrane transport. RT-qPCR of high- and poor-quality sperm samples allowed showing that the expression of some genes, such as *AURKA*, *HDAC4*, *CFAP46*, *SPATA18*, *CACNA1C*, *CACNA1H*, *CARHSP1*, *CCDC60*, *DNAH2*, and *CDC88B*, have different expression levels according to sperm morphology. In conclusion, the present study shows a strong correlation between morphology and gene expression in the spermatozoa and provides a biomarker panel for sperm analysis during ART and a new tool to explore male infertility.

## 1. Introduction

**Figure 1:**
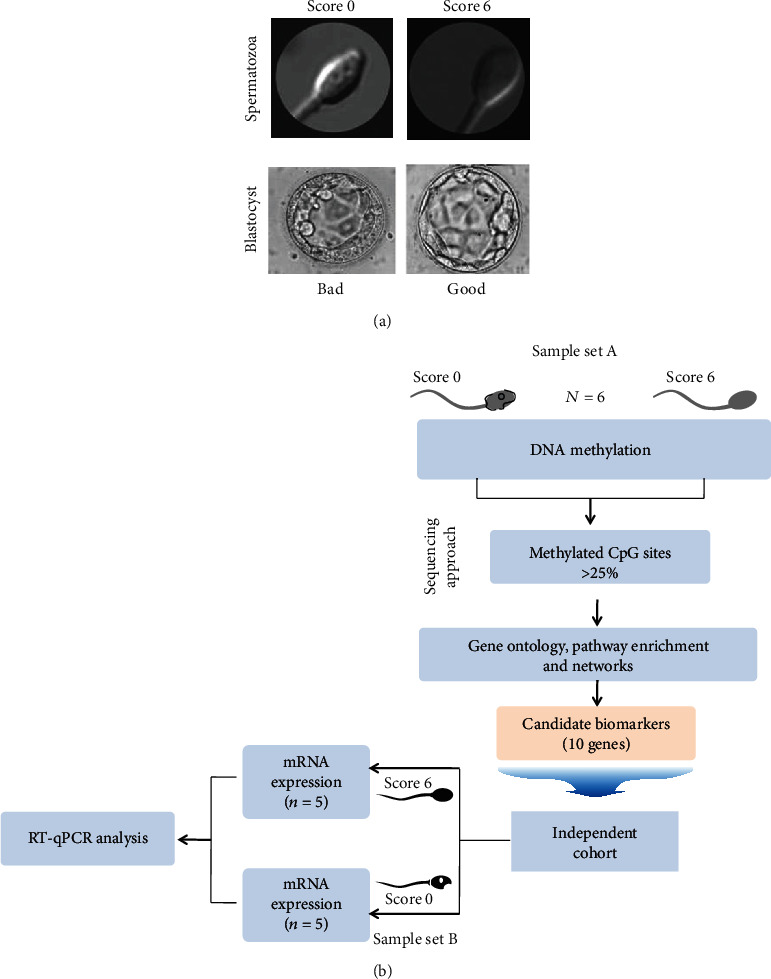
Study design. (a) Morphological criteria are used to score spermatozoa at high magnification (6100x) and to assess blastocyst quality under an inverted microscope. (A) Representative images of a spermatozoon with “score 0” (top) and the bad-quality blastocyst obtained by ICSI using this spermatozoon. (B) Representative images of a spermatozoon “score 6” and the good-quality blastocyst obtained. (b) Simplified flowchart of the strategy to identify candidate biomarkers of good-quality spermatozoa. DNA from sperm samples (*n* = 3 with “score 0” and *n* = 3 with “score 6”) is sequenced to identify an initial set of genes that are differentially methylated in spermatozoa with good (score 6) and poor (score 0) morphology. The correlation between the expression profiles of candidate genes and spermatozoon morphology is then evaluated by reverse transcription quantitative polymerase chain reaction (RT-qPCR) using RNA from an independent set of sperm samples (*n* = 5 with “score 0” and *n* = 5 with “score 6”) from different patients with infertility.

Male infertility affects roughly 30 million men worldwide and contributes to 50% of all infertility cases (15% of the 60 to 80 million couples trying to conceive) [1, 2]. Different causes of male infertility have been identified (e.g., hormonal, mechanical, postinfectious, chromosomal, and genetic) [3, 4], but in about 50% of cases, the origin remains unknown. Routine sperm analysis includes the evaluation of sperm volume, pH, concentration, motility, vitality, and morphology [5]. The current treatment for male infertility associated with abnormal sperm parameters is fertilization by intracytoplasmic sperm injection (ICSI) [6] after selection of motile and morphologically normal spermatozoa. Indeed, the injection of individual sperm cells with abnormal or subnormal morphology can reduce fertilization and implantation rates [7]. However, some spermatozoa that appear morphologically normal at ×200 or ×400 magnification present abnormalities when examined at higher magnification (×6100). Therefore, in 2009, we introduced a new classification of living spermatozoa with a scoring scale ranging from 0 to 6 based on strict morphologic criteria [8]. Briefly, the spermatozoa with a total score of 6 points display normal head shape (normal head, with symmetrical nuclear no extrusion and/or no invagination of the nuclear membrane = 2 points) without any vacuole (3 points), and normal base (the third inferior part of the sperm head to the neck where the centrosome is localized = 1 point). The spermatozoa with a total score 0 (0 point) display head abnormalities, vacuoles, and abnormal base (Figure 1(a)). Fertilization rates and number of good-quality blastocysts at day 5 (according to Gardner's classification [9]; Figure 1(a)) are significantly higher when using the spermatozoa with score 6 (6 points) [8, 10]. Similarly, other studies showed higher implantation and pregnancy rates and lower miscarriage rates when performing ICSI with the spermatozoa of good morphology selected by microscopy analysis at high magnification [11, 12]. Moreover, a meta-analysis suggests an increased risk of birth defects in children conceived by ICSI compared with those born after *in vitro* fertilization (IVF) or spontaneous conception [13]. However, the rate of major malformations is significantly reduced when ICSI is performed after sperm selection at very high magnification [14, 15], highlighting the importance of sperm selection. In addition, the approaches currently used for sperm selection are still not fully adequate [16], emphasizing the need of a strategy that takes into account not only morphological features but also functional. For instance, genome alterations, chromatin structure, DNA fragmentation, and epigenetic profile (e.g., DNA and histone methylation patterns) of the sperm contribute to proper embryo development and healthy live birth [17]. The spermatozoa play a crucial role by delivering a novel epigenetic signature to the egg [18]. In previous studies, we evaluated the relationship between sperm head morphology at high magnification and its chromatin/DNA content in sperm samples from men harboring a reciprocal or a Robertsonian translocation. However, we did not detect any relationship between high-magnification morphology and balanced/unbalanced chromosomal content [19, 20]. This could be related to the fact that in carriers of chromosomal rearrangements, all spermatozoa, and not only those with chromosomal unbalance, display an abnormal nuclear chromosomal architecture [21]. We also showed that score 0 spermatozoa are associated with high levels of sperm chromatin decondensation, but not with DNA fragmentation [22, 23]. Other studies showed a correlation between vacuole size and DNA fragmentation [24–31]. All these studies emphasize the evident correlation between sperm head morphology at very high magnification and its chromatin status [32] and highlight the heterogeneity of spermatozoa from the same ejaculate that consequently will not give the same outcome after oocyte fertilization. They carry the same genetic DNA information, but in a differently packaged chromatin with different degrees of compaction and possibly different degrees of protection against external microenvironment. Moreover, variations in the DNA/histone methylation profile among spermatozoa might have crucial consequences for gene expression and possibly for early embryo development and ART outcome [33, 34]. In agreement, we previously reported lower DNA methylation levels in the spermatozoa with score 6 than with score 0 from the same sample. This allows selecting the spermatozoa without abnormal DNA methylation levels and thus reducing the risk of birth defects [35]. It is generally accepted that the incidence of major malformations is lower after spontaneous conception than after ART [36]. All these data establish correlations between head morphology at high magnification and chromatin/DNA status and suggest that there may be also a link with gene expression.

The aim of the present study was to (i) compare the DNA methylation profile, assessed by whole-genome sequencing, in the spermatozoa classified according to their high-magnification morphology (score 6 versus score 0) to identify genes that are differentially methylated in these two categories and (ii) to compare the expression of some of these differentially methylated genes in the spermatozoa classified according to their morphology score (score 6 versus score 0). As coding transcripts contribute to the production, morphology, and function of viable human sperm, we hypothesized that their methylation pattern and expression levels in morphologically scored spermatozoa could be used as biomarkers to select high-quality sperm in term of ART outcome.

## 2. Material and Methods

### 2.1. Sample Collections and Sperm Parameters

The study protocol was approved by the local ethics committee, the members of which are part of the Institutional Review Board (IRB) of the Société d'andrologie de langue Française (IORG0010678). The study protocol was carried out in the ART Unit of the Drouot Laboratory, Paris, France. All participants signed an informed consent form before inclusion in the study. They were informed that after the clinical tests, the semen sample would be analyzed at high magnification and using molecular biology approaches. The patients' confidentiality was ensured by data anonymization before analysis. This analysis did not lead to any additional costs for the patients and did not affect their treatment in any way.

For this study, 16 couples were enrolled in our IVF unit for different (female or/and male) infertility problems. In these 16 couples, men were divided in two groups on the basis of the percentage of sperm with score 6 (good sperm quality) and score 0 (bad sperm quality): (i) one group of men with good sperm quality (5%-15% of spermatozoa with score 6 and less than 30% of spermatozoa with score 0). In an internal study of 1000 ejaculates with good sperm parameters, not more than 15% of spermatozoa had score 6; (ii) the other group of men with bad sperm quality (90% of spermatozoa with score 0 and 0% with score 6).

The analysis concerned 16 fresh sperm samples from 16 men (39 ± 5.6 years) selected according to the sperm morphological scores: 6 samples (three with score 6 and three with score 0) were used for the DNA methylation analysis by whole-genome bisulfite sequencing to select candidate genes and 10 (five with score 6, and five with score 0) for the gene expression analysis by RT-qPCR. The parameters of these 16 samples are summarized in Supplementary Table S1.

### 2.2. Sperm Preparation

Motile spermatozoa were isolated and purified by bilayer concentration density gradient in conical tubes containing 45% and 90% of ISolate Sperm Separation Medium (Cat. no. 99264; Fujifilm Irvine Scientific, Santa Ana, CA, USA). Tubes were centrifuged at 300×g for 15 min. Then, the supernatant was discarded, and sperm pellets were washed in the modified Human Tubal Fluid (mHTF) medium (Cat. no. 90126; Fujifilm Irvine Scientific, Santa Ana, CA, USA) and centrifuged at 600 g for 10 min. Pellets were then resuspended in 500 *μ*l mHTF medium, counted under a light microscope high magnification, and selected according to concentration of the score. All motile spermatozoa were sorted at high magnification (×6100) according to strict morphological criteria [5] combined with the previously described scoring scale. The 16 samples were frozen and stored at -80°C for DNA isolation.

### 2.3. Whole-Genome Bisulfite Sequencing and Methylation Analysis

The SeqCap Epi Enrichment System (ROCHE NimbleGene), a solution-based capture method, was chosen because it allows the enrichment of bisulfite-converted DNA in a single tube and sequenced on the NextSeq 500 platform (Illumina) according to manufacturing protocol. Raw data were mapped to the human reference genome (*Homo sapiens* genome build GRCh37 (hg19) with the BSMAP (v 2.89) software, using the methylKit R package [37]. Potential differentially methylated regions were identified with strict filters (*q* value < 0.01 and methylation difference percentage > 25%).

### 2.4. Gene Ontology Analysis and Functional Enrichment

Targeted gene function was assessed with Gene Ontology (GO), the PANTHER tool (http://pantherdb.org), and the Genomatrix software suite (Genomatix Software GmbH, Munich, Germany). The OmicsNet tool was used for network creation and visual exploration [38]. Data (differentially methylated genes) were integrated with molecular interactions using the ingenuity pathway analysis (IPA) software application (http://www.ingenuity.com). Each gene symbol was mapped to the corresponding gene object in the Ingenuity Pathways Knowledge Base. Gene networks were algorithmically generated based on their connectivity and assigned a score. The score is a numerical value used to rank networks according to their relevance to the genes in the input dataset but may not be an indication of the network quality or significance. The score takes into account the number of focus genes in the network and the network size to approximate how relevant the network is to the original list of focus genes.

### 2.5. RNA Isolation and Relative Gene Expression Analysis

RNA was extracted from sperm samples with the miRNeasy Kit (QIAGEN) following the manufacturer's protocol. cDNA templates were prepared by reverse transcription (RT) using the ReadyScript cDNA Synthesis Kit (Sigma-Aldrich) starting from 100 ng total RNA following the manufacturer's protocol. cDNA templates were 1 : 11 diluted in 0.1x TE before analysis by quantitative PCR (qPCR) with TaqMan Gene Expression Assays (Applied Biosystems) on a LightCycler 480 (Roche Diagnostics). Two housekeeping genes were used as reference: *β*2 microglobulin (*B2M*) and protamine 1 (*PRM1*). The *M* values (i.e., the average gene expression stability) [39] were determined, and the *M* value cut-off for the reference genes was 0.25. The following ten genes (TaqMan Gene Expression Assay ID number) were selected for RT-qPCR analysis based on their potential function: *AURKA* (Hs01582072_m1), *HDAC4* (Hs01041648_m1), *SPATA18* (Hs01102818_m1), *CCDC60* (Hs00905317_m1), *CACNA1H* (Hs01103527_m1), *CCDC88B* (Hs00989955_g1), *DNAH2* (Hs01044842_m1), *CACNA1C* (Hs00167681_m1), *CARHSP1* (Hs00183933_m1), *CFAP46* (Hs00929098_m1), *PRM1* (Hs00358158_g1), and *B2M* (Hs00187842_m1). All qPCR assays were carried out in 384-well plates in three technical replicates in 10 *μ*l final reaction volume using the TaqMan Fast Advanced Master Mix (2x) and the following cycling conditions: 95°C for 20 seconds (enzyme activation), followed by 45 cycles of denaturation at 95°C for 3 seconds, annealing at 60°C for 30 seconds, and extension with fluorescence measurement. The relative gene expression was calculated with the 2^-*ΔΔ*Cp^ method [40]. Cp indicates the cycle threshold, i.e., the fractional cycle number when the fluorescent signal reaches the detection threshold. The normalized *Δ*Cp value of each sample was calculated using the reference gene values with a Cp variation < 1 in all experiments.

### 2.6. Statistical Analyses

Unless otherwise indicated, differences were considered significant when the unpaired two-tailed Bonferroni-adjusted *p* value (*Q*) was < 0.05. Fisher's exact test and unadjusted *p* values were used for the IPA and motif enrichment analyses and the Mann–Whitney test [41] to compare the RT-qPCR results (Cp values), in order to avoid reduction in variance and introducing dependence among the normalized values.

## 3. Results

The objective of the present study was to identify molecular biomarkers in human spermatozoa that correlate with a good morphological aspect (i.e., score = 6). To this aim, gene methylation/expression analyses were performed using a combination of DNA-seq and RT-qPCR methods (Figure 1(b)). This allowed identifying ten genes, the expression level of which was correlated with spermatozoa morphology. These genes might be used as biomarkers of sperm quality or as pharmacological targets to improve the fertilization potential of spermatozoa.

### 3.1. Identification of Regions with Differential DNA Methylation in Spermatozoa with Good and Poor Morphology by Whole-Genome Bisulfite Sequencing

**Figure 2:**
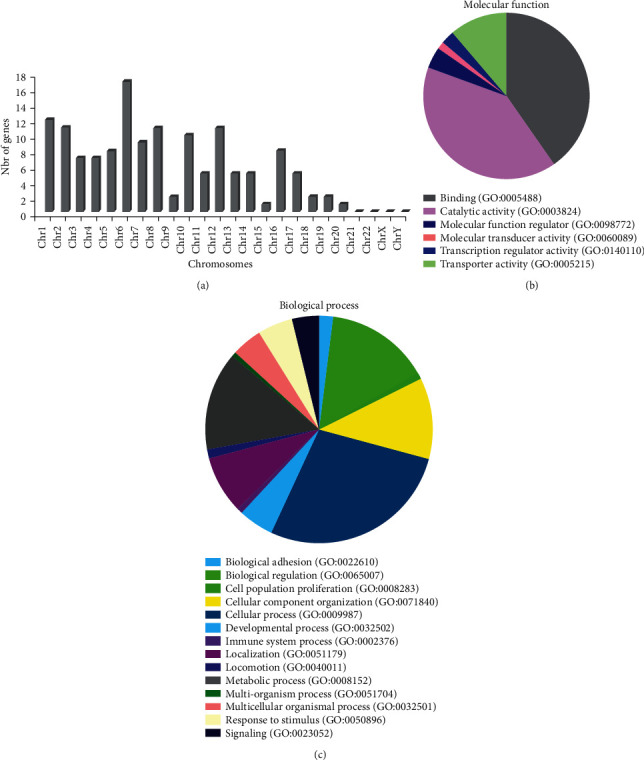
Analysis of the differentially methylated genes. (a) Distribution of differentially methylated genes between score 0 and score 6 sperm samples in the human chromosomes. (b) Gene Ontology (GO) classification of the differentially methylated genes in molecular function categories. (c) GO classification of the differentially methylated genes in biological process categories. The two pie charts were generated with the PANTHER tool.

To determine the global DNA methylation profile of six human sperm samples (three with high-magnification morphology score 0 and three with score 6), first bisulfate conversion, DNA-sequencing analysis, and CpG methylation profiling at the single-base resolution were performed (Figures 1(a) and 1(b)). The genome-wide DNA methylation analysis identified 17,544 methylation-variable positions with genome-wide significance (adjusted *p* < 0.05). By using a strict filter (*q* value < 0.01 and percentage of methylation difference between the score 0 and score 6 samples >25%) to detect potentially differentially methylated regions, 746 positions were identified, including 138 known genes (Supplementary Table S2) and 308 genomic loci. Differentially methylated CpG bases were detected in all chromosomes, except chromosomes 21, 22, X, and Y (Figure 2(a)). The list of differentially methylated genes (sperm methylation signature) included genes encoding proteins associated with sperm motility, flagellar assembly, and spermatogenesis (*DNAH2*, *CFAP46*, and *SPATA18*), coiled-coil domain (*CCDC88B* and *CCDC60*), many channel-mediating calcium and sodium entry (*CACNA1C*, *CACNA1H*, *CACNA2D4*, *TRPM3*, *SCN8A*, and *ANO2*), calcium-regulated proteins (*CARHSP1*, *ATXBP5*, and *SMOC2*), histones (*HDAC4*, *JMJD1C*, and *SMYD3*), E3 ubiquitin-protein ligase (*ZNFR4*, *CHFR*, *PARK2*, *MARCH6*, *SPSB1*, and *HECTD2*), linked to the cytoskeleton molecular organization (*SNTG2*, *PHACTR1*, *SYNE1*, and *DLGAP2*), transporter activity (*SLC2A1*, *SLC18A8*, *SLC35F3*, and *CLCN7*), ATPase activity (*ATP6V0A4*, *PFKP*, *CARKD*, and *NKAIN3*), zinc finger proteins (*ZNF239*, *ZFYVE28*, and *DPF3*), transmembrane proteins (*TMEM117*, *TMTC4*, and *LRTM2*), transcription factors (*SOX6* and *GATA4*), and genes associated with mitochondria (*NDUFB4*, *TOP1MT*, *SDHA*, and *ADHFE1*).

### 3.2. Functional Properties of the Genes Enriched in the Sperm Methylation Signature

Gene ontology (GO) annotations were used to identify the potential biological processes and functional properties of the genes included in the sperm methylation signature (Figures 2(b) and 2(c)). The top “biological processes” terms were locomotion (GO: 0040011), cellular process (GO: 0009987), cell population proliferation (GO: 0008283), metabolic process (GO: 0008152), cellular component organization (GO: 0071840), and response to stimulus (GO: 0050896). The most enriched “molecular function” terms were binding (GO: 0005488), catalytic activity (GO: 0003824), and transporters (GO: 0005215). The genes of the sperm methylation signature were also analyzed using the Genomatix Genome Analyzer to identify the most relevant molecular and cellular functions (Table 1). The functional categories identified were highly relevant to sperm function, such as calcium channel activity (*p* = 1.12*E*^−03^), enzyme binding (*p* = 2.56*E*^−03^), ATP-dependent microtubule motor activity (*p* = 3.41*E*^−03^), protein binding (*p* = 8.37*E*^−03^), passive transmembrane transporter activity (*p* = 8.64*E*^−03^), and cytoskeletal protein binding (*p* = 8.66*E*^−03^).

### 3.3. Regulatory Roles and Potential Networks Associated with the Identified Genes

**Figure 3:**
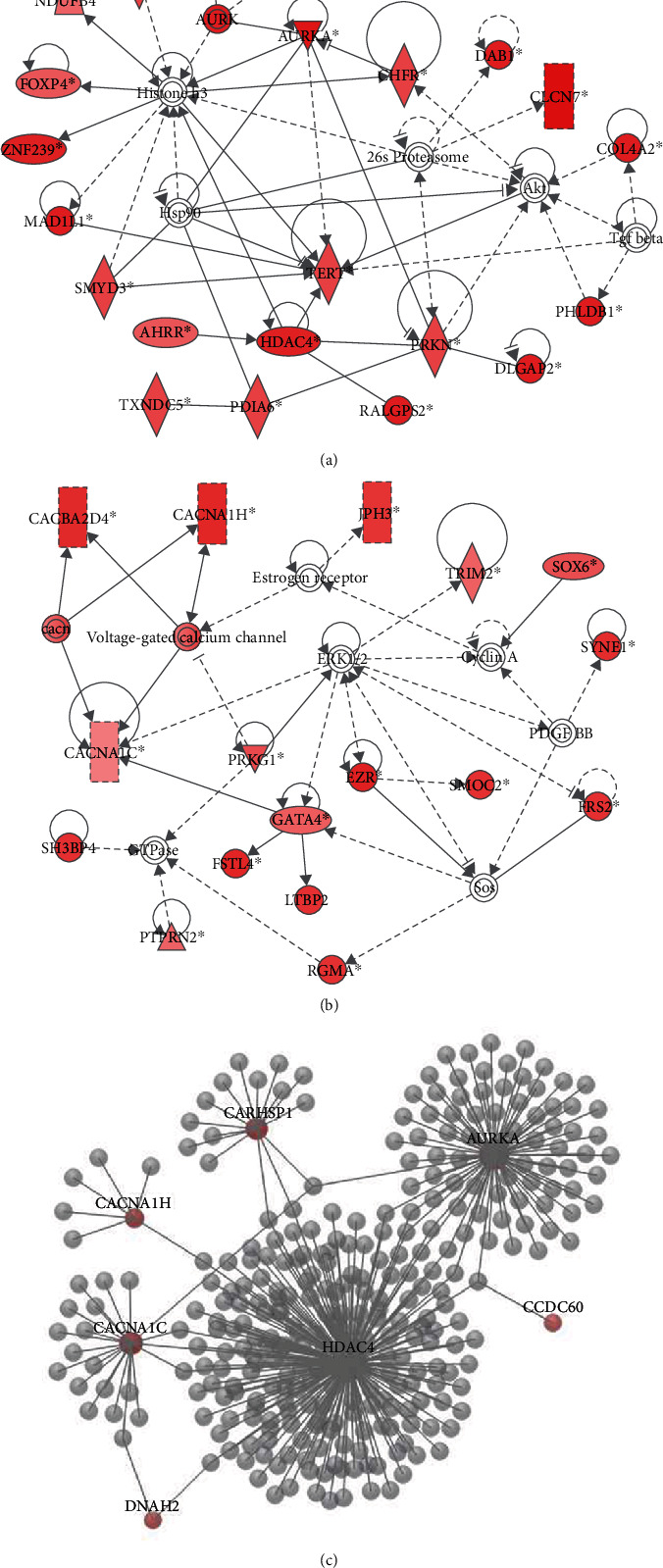
Top-ranked functional networks of the differentially methylated genes. (a) Top network identified by ingenuity pathway analysis (IPA) of differentially methylated genes related to cell cycle, cellular assembly, and organization. (b) Top network identified by IPA of differentially methylated genes related to organ development and function. Colored nodes indicate differentially methylated genes. Noncolored nodes were proposed by IPA and suggest potential targets functionally coordinated with the differentially methylated genes. Dashed lines represent indirect relationships, and solid lines indicate direct molecular interactions. In each network, edge types are indicatives: a line without arrowhead indicates binding only; a line finishing with a vertical line indicates inhibition; a line with an arrowhead indicates ‘acts on.' (c) Protein-protein interaction network of selected differentially methylated genes. Using the OmicsNet database, six genes (*AURKA*, *HDAC4*, *CARHSP1*, *CACNA1H*, *CACNA1C*, and *DNAH2*) were used to construct a top-ranked functional protein-protein interaction network.

Then, IPA was used to explore the putative functions of genes and networks of the sperm methylation signature. This analysis identified two top networks (Figures 3(a) and 3(b)). Each network showed interactions with major signaling pathway molecules, including histones, *AKT*, *ERK1/2*, and cyclins. Functions associated with these networks included cell cycle, cellular assembly and organization, and organ development and function. The aurora kinase A *AURKA*-centered network (Figure 3(a)) functionally interacted with spermatogenesis-associated 18 (*SPATA18*), *CHFR*, *INSYN2A*, *PRKN*, *TERT*, and histones, forming a tightly connected network. Histone deacetylase 4 *(HDAC4*) also was related to histones and displayed direct interaction with *TERT*, *AHRR*, *PRKN*, and *RALGPS2*. The second network (Figure 3(b)) showed interactions with voltage-gated calcium channel genes (*CACNA1H*, *CACNA1C*, and *CACNA2D4*) and also between calcium channel components and *PRKG1*, *GATA4*, and *ERK1/2*, suggesting an operative role of channels that mediate calcium entry in the spermatozoa. To establish functional links among these molecules, the protein-protein interaction network was constructed. It showed that AURKA was highly connected with HDAC4 (Figure 3(c)) and gave results very similar to those obtained by the IPA function and pathway analysis. Dynein axonemal heavy chain 2 (DNAH2) and coiled-coil domain containing 60 (CCDC60), which are involved in ciliogenesis, were included in this network. In addition, most of the nodes forming this network were associated with proteins implicated in sperm motility and flagellar assembly (for instance, CACNA1C and CACNA1H) and calcium regulated heat stable protein 1 (CARHSP1).

### 3.4. Relationship between Gene Expression Pattern and Sperm Morphology

**Figure 4:**
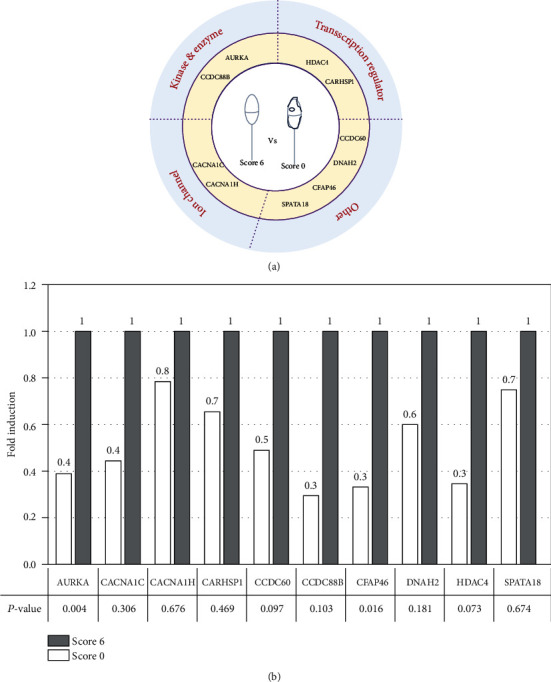
Relative expression level of 10 genes that are differentially expressed in score 0 and score 6 spermatozoa. (a) Graphical representation of the gene types. (b) Gene expression was compared between score 0 (white) and score 6 (black; reference, set to 1) sperm samples by RT-qPCR analysis. *p* value were calculated with the Mann–Whitney test.

Then, to evaluate the relationship between gene expression and sperm morphology, the expression of ten genes included in the sperm methylation signature (*HDAC4*, *AURKA*, *CFAP46*, *DNAH2*, *CCDC88B*, *CACNA1C*, *CACNA1H*, *SPATA18*, and *CARHSP1*) was quantified in 10 sperm samples with poor and good morphology (*n* = 5 with score 0 and *n* = 5 with score 6, respectively) (Figure 1(b)). These genes were chosen because they participate in the regulatory mechanisms of physiological processes during spermatogenesis, such as cell cycle, locomotion and cell motility, cellular assembly and organization, and the calcium activation pathways, according to the IPA (Figure 4(a)). To increase the robustness of the RT-qPCR experiments, two reference genes (*B2M* and *PRM1*) were used to obtain the average expression stability of the reference genes using the *M* value method [39]. The two genes had *M* values < 0.2. The RT-qPCR results showed that the expression level of the 10 genes was higher in the morphologically good samples than in the morphologically poor samples (Figure 4(b)). This suggests that their expression is positively correlated with sperm morphology and that these genes might be candidate biomarkers of sperm quality and health to be used during the selection of spermatozoa for ICSI.

### 3.5. Expression in Various Human Tissues

**Figure 5:**
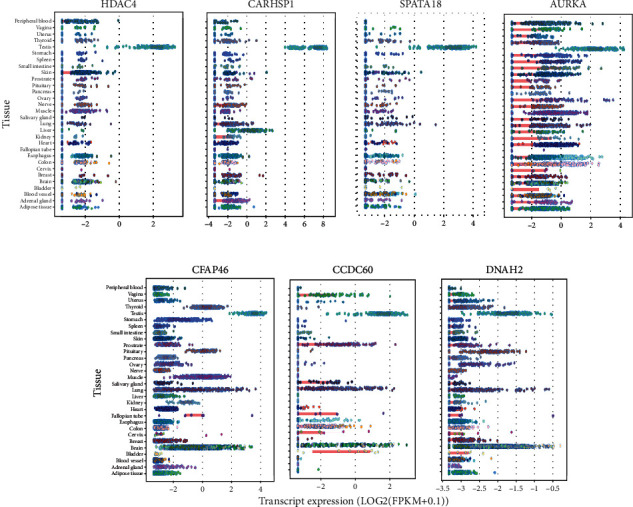
Expression profile of candidate genes in different human tissues. Expression values (in Log2 (RPKM)) of *HDAC4*, *CARHSP1*, *SPATA18*, *AURKA*, *CCDC60*, *DNAH2*, and *CFAP46* in 30 tissues from the Genotype-Tissue Expression (GTEx) consortium. For each gene, the colored circle belonging to each tissue indicates the valid RPKM value of all samples in the tissue. RPKM: reads per kilobase of transcript per million mapped reads.

To determine whether these ten genes were testis-specific, their expression was analyzed in normal tissues using RNA-seq data (expressed as log reads per kilobase of transcript per million mapped reads (RPKM)) for 30 tissue types from the Genotype-Tissue Expression (GTEx) repository [42, 43]. This analysis showed that *HDAC4*, *CARHSP1*, *SPATA18*, and *AURKA* were strongly expressed in testis tissue compared with other tissues (Figure 5). In contrast to *HDAC4*, *CARHSP1*, and *SPATA18*, *AURKA* is also highly expressed in the ovary, as well as in the lung, esophagus, and colon. *CFAP46*, *CCDC60*, and *DNAH2* were strongly expressed in tissues that contain cilia, such as the testis, lung, and brain (Figure 5). The expression of *CCDC88B* and *CACNA1H* in the testis is rather low compared to most of the other tissues as shown in (Supplementary Figure S1). Moreover, no difference in expression was observed for *CACNA1C* in the different tissue samples *AURKA*, *HDAC4*, and *CARHSP*1 could play a role during the maternal to embryonic genome transition and in embryonic genome activation [44]. Specifically, *AURKA* could be involved in cell division up to the embryonic genome activation. Future studies should determine whether alterations of *AURKA* and *SPATA18* gene expressions affect human early embryo development.

## 4. Discussion

Male infertility is commonly associated with high rates of sperm DNA damage, and the quality of human sperm is one of the main determinants of ART success. Our previous studies focused on assessing the relationship between sperm head morphology and DNA methylation levels through detection of 5-methylcytosine residues by fluorescence microscopy [35]. Here, we used whole-genome bisulfite sequencing and methylation analysis to investigate the methylome of morphologically scored spermatozoa. First, we identified regions that are differentially methylated between score 6 and score 0 spermatozoa (good and poor morphology, respectively) and investigated their function and regulatory networks. We found that methylation differences varied among chromosomes. Chromosome 6 had the highest number of differentially methylated regions and chromosomes 21, 22, X, and Y had none. This might be related to the gene distributions in the different chromosomes. Additional studies are needed to understand the accurate mechanisms of epigenetic regulation.

Then, we investigated the relationship between the gene expression level and sperm morphology. We found that the expression of some genes with functions related to sperm motility, flagellar assembly, and spermatogenesis was affected, including spermatogenesis-associated 18 (*SPATA18*), cilia- and flagella-associated protein 46 (*CFAP46*), and dynein axonemal heavy chain 2 (*DNAH2*). *SPATA18* transcription in mammalian seminiferous tubules is induced by p53 [45] that is implicated in meiosis during spermatogenesis and guarantees the appropriate quality of mature spermatozoa [46, 47]. *SPATA18* transcriptional regulation is necessary for spermatogenesis progression [45]. *CFAP46* is part of the central apparatus of the cilium microtubule-based cytoskeleton. In humans, alterations of cilia- and flagella-associated protein family genes (e.g., *CFAP43*, *CFAP44*, and *CFAP251*) have been associated with the multiple morphological abnormalities of the flagella (MMAF) syndrome [48]. *DNAH2* is essential for human ciliary function. The spermatozoa from patients with MMAF and *DNAH2* mutations display loss of motility [49], and patients with *DNAH2* gene variations show defects of the MMAF phenotype [50]. Although *CFAP46* and *DNAH2* are expressed primarily in testis, here, we found that they are expressed also in other tissues, such as the brain and lungs. Several studies have characterized ion channels in the sperm from the fertile and unfertile patients [51]. It has become clear that ion channel activities play a key role in sperm function. Our sequencing data revealed that many genes encoding channels that mediate calcium and sodium entry are differentially methylated in spermatozoa with good and poor morphology. Moreover, the genes encoding voltage-dependent L-type calcium channel subunit alpha-1C and calcium channel subunit alpha-1H *(CACNA1C* and *CACNA1H*) and calcium-responsive heat-stable protein 1 (*CARHSP1*) are more strongly expressed in score 6 than in score 0 spermatozoa. The implication of *CACNA1C* and *CACNA1H* in many calcium-dependent processes (e.g., muscle contraction, neurotransmitter release, cell motility, and cell death) and their association with different diseases [52–54] suggest that their dysfunction could significantly affect sperm function. The importance of the RNA-binding protein CARHSP1 (also called CRHSP-24) during spermatogenesis was previously reported in mice [55].

Our study also showed that the genes encoding aurora kinase A (*AURKA*) and histone deacetylase 4 (*HDAC4*) are differentially expressed in score 6 and score 0 spermatozoa and that they represent excellent candidate biomarkers. Aurora kinase A is implicated in the proper execution of various mitotic events, including centrosome maturation, separation, spindle formation, and mitotic entry [56]. Besides its function in dividing spermatogonia and spermatocytes, this kinase is involved in sperm development and motility, which are critical for male fertility [57]. Its activation involves many proteins that together affect primary cilia disassembly [58]. HDAC4, a class II histone deacetylase, acts by forming large multiprotein complexes and plays an important role in transcriptional regulation, cell cycle progression, and developmental events. HDAC6 (another class II histone deacetylase) promotes microtubule destabilization in vivo [59] and is phosphorylated in the presence of aurora kinase A [58]. Additionally, aurora kinase A activation in ciliary disassembly requires its interaction with Ca^(2+)^ and calmodulin [60], leading to transient Ca^2+^ signals in ciliary disassembly via the AURKA–HDAC6 signaling cascade [60, 61].

Remarkably, analysis of the *AURKA* interactome highlighted interactions with *HDAC4* and connection with calcium channel subunit alpha *(CACNA1C* and *CACNA1H*). This suggests that a defect in any components of the calcium flux- aurora kinase A-histone deacetylase signaling cascade might impact sperm function and consequently fertility. Additional research is required to understand the importance of *AURKA*, *HDAC4*, and channel-mediating calcium entry in spermatogenesis. Aurora kinase A is also implicated in the establishment of the achromatic spindle allowing chromosome migration for cell division during embryo development [62, 63]. This could explain the impaired development (slow kinetic, fragmented embryos with irregular blastomeres, and absence of expanded blastocysts) after ICSI with scores 0 spermatozoa. Moreover, we found that *AURKA* expression is higher during the early embryo cleavage stages, but then declines between the 4-cell and 8-cell stage, concomitantly with maternal genome degradation and embryonic genome activation. This suggests that *AURKA* might influence the maternal-embryonic genome transition. Future studies should determine whether altered *AURKA* expression in the spermatozoa affects human early embryonic development.

## 5. Conclusion

We found a significant differential DNA methylation and expression of many genes in sperm with poor and good morphology obtained from patients referred for ICSI for male infertility. This study provides a new panel of genes (A*URKA*, *HDAC4*, *CACNA1C*, *CACNA1H*, *CARHSP1*, *CFAP46*, *SPATA18*, *CCDC60*, *DNAH2*, and *CDC88B*) that could be used as biomarkers to assess sperm quality. Despite the study limitations (i.e., low sample size and the fact that samples used for DNA and RNA sequencing were not from the same patient), we identified a set of genes that might be candidate biomarkers of sperm morphology and new drug targets for the treatment of spermatozoa defect. Before any routine use, a large cohort of sperm samples should be analyzed by high-magnification microscopy and RT-qPCR approaches. Our work suggests that rapid molecular analysis of few genes in sperm samples might contribute to the selection of good-quality spermatozoa and could avoid chaotic or abnormal early embryo development after ICSI. The envisioned use of the RT-qPCR-based transcriptomic analysis to identify the spermatozoa with good gene expression profiles is described in Supplementary Figure S2.

## Figures and Tables

**Table 1:** Functional classification of the methylated genes using Genomatix software.

Functional categories	GO term ID	*p* value	List of genes
Calcium channel activity	GO:0005262	1.12*E* − 03	CACNA1H, CACNA1C, CACNA2D4, TRPM3, JPH3
Dolichyl-phosphate-mannose-protein mannosyltransferase activity	GO:0004169	1.37*E* − 03	TMTC4, POMT2
Enzyme binding	GO:0019899	2.56*E* − 03	SLC9A3R2, ATP6V0A4, EXOC2, FRS2, EZR, CARHSP1, STXBP5, PHACTR1, CNST, RALGPS2, SH3BP4, AURKA, SMYD3, PRKN, SLC2A1, DENND3, RASGEF1A, JAKMIP3, MARCHF6, HDAC4, SYNE1, ARFGEF3, PSD3, MCF2L, GATA4
Coreceptor activity	GO:0015026	2.83*E* − 03	GPC6, CD80, RGMA
ATP-dependent microtubule motor activity	GO:1990939	3.41*E* − 03	KIF26A, DNAH2, KIF17
Isomerase activity	GO:0016853	4.29*E* − 03	TXNDC5, NAXD, QSOX1, TOP1MT, PDIA6
Ubiquitin-specific protease binding	GO:1990381	5.61*E* − 03	PRKN, MARCHF6
Tubulin binding	GO:0015631	5.95*E* − 03	KIF26A, EZR, CCDC88B, TBCD, PRKN, JAKMIP3, KIF17
Oxidoreductase activity, acting on a sulfur group of donors	GO:0016667	6.09*E* − 03	QSOX1, PDIA6, NXN
ARF guanyl-nucleotide exchange factor activity	GO:0005086	6.25*E* − 03	ARFGEF3, PSD3
GTPase binding	GO:0051020	7.52*E* − 03	EXOC2, STXBP5, RALGPS2, SH3BP4, DENND3, RASGEF1A, ARFGEF3, PSD3, MCF2L
Protein binding	GO:0005515	8.37*E* − 03	TRIM2, CACNA1H, SPSB1, KIF26A, SLC9A3R2, ESPNL, AUTS2, TXNDC5, CACNA1C, TCAF2, ZFYVE28, SPON2, ATP6V0A4, MAD1L1, EXOC2, FRS2, DPF3, ERGIC1, SPATA18, CUX1, EZR, COLEC11, RPA3, GPC6, AHRR, MFAP3L, CARHSP1, DNAH2, ZNRF4, PFKP, BANP, CCDC88B, SCN8A, STXBP5, PHACTR1, CNST, RALGPS2, NAXD, TBCD, PRKG1, JMJD1C, USP10, LRTM2, ST8SIA5, CCDC60, DLGAP2, SH3BP4, AURKA, TG, LTBP2, SMYD3, PRKN, COL4A2, SLC2A1, IGHMBP2, DENND3, SDHA, HMGB4, CHFR, MPHOSPH10, SNTG2, RASGEF1A, JAKMIP3, PDIA6, PHLDB2, MARCHF6, FRK, HDAC4, TBL3, TLE1, SYNE1, ARFGEF3, PSD3, KIF17, ZNF239, FOXP4, MCF2L, FSTL4, ANO2, SOX6, GATA4, AGPAT4, CD80, SPPL2B, TERT, DAB1, RGMA
Passive transmembrane transporter activity	GO:0022803	8.64*E* − 03	CACNA1H, CACNA1C, CLCN7, CACNA2D4, SCN8A, TRPM3, JPH3, ANO2
Cytoskeletal protein binding	GO:0008092	8.66*E* − 03	KIF26A, ESPNL, CACNA1C, EZR, CCDC88B, STXBP5, PHACTR1, TBCD, PRKN, SNTG2, JAKMIP3, SYNE1, KIF17

## Data Availability

The data used to support the findings of this study are included within the article and available from the corresponding author upon request.

## Supplementary Materials

Supplementary 1 Supplementary Table S1 Sample collected and sperm parameters. Supplementary Table S2: list of differentially methylated genes (*n* = 138) between score 6 and score 0 sperm samples

Supplementary 2 Supplementary Figure S1: expression values (in Log2 (RPKM)) of *CCDC88B*, *CACNA1H*, and *CACNA1C* in 30 tissues from the Genotype-Tissue Expression (GTEx) consortium. Supplementary Figure S2: envisioned sperm test for male infertility in ART.
