# Supplementary figures and images for: Molecular Profiling of Spermatozoa Reveals Correlations between Morphology and Gene Expression: A Novel Biomarker Panel for Male Infertility
Source: Biomed Res Int. 2021 Sep 18;2021:1434546. doi: 10.1155/2021/1434546 (PMC8485144; doi:10.1155/2021/1434546)

Supplementary Figure S1

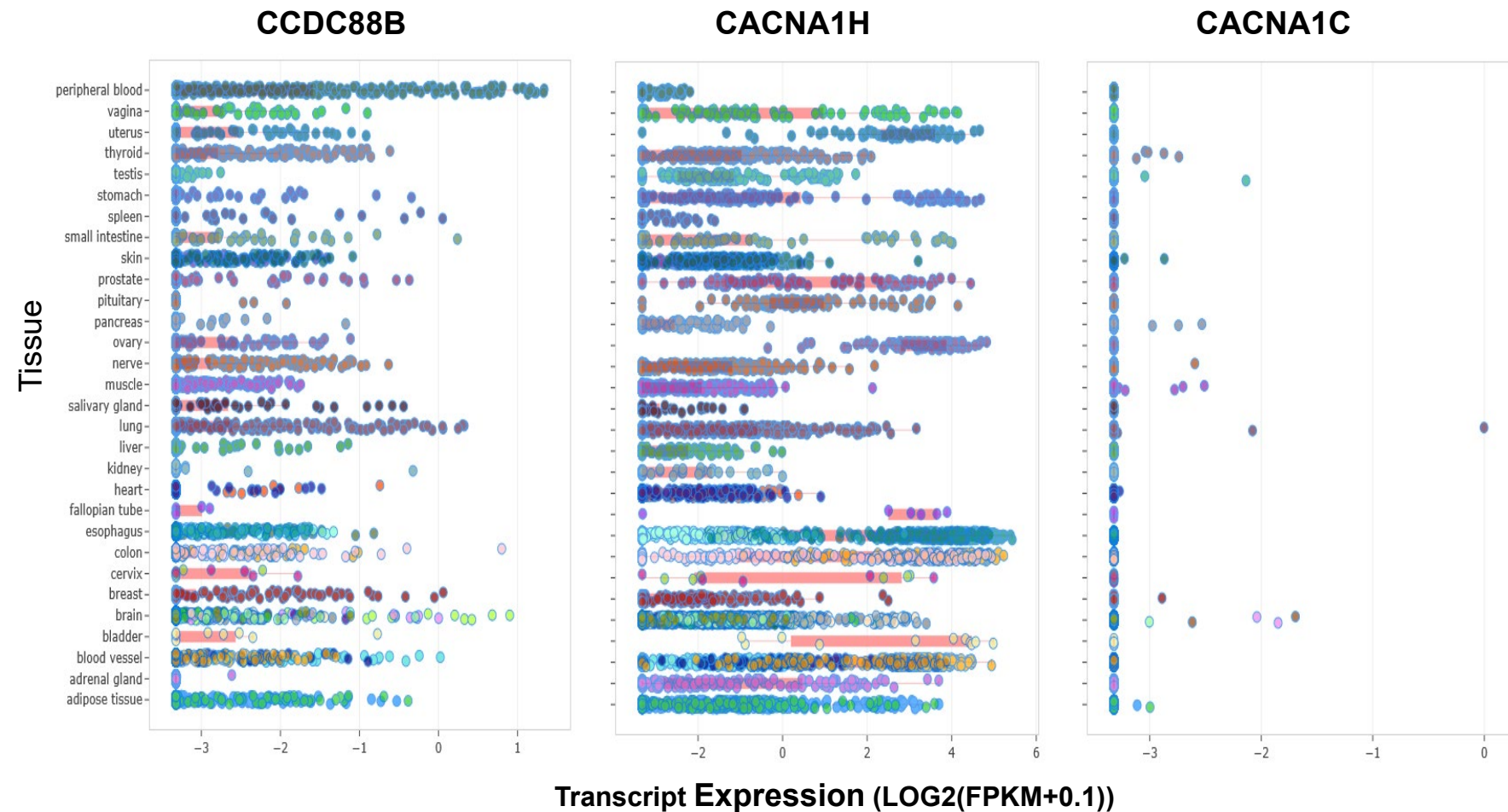

Supplement: Supplementary 1 — Supplementary Table S1 Sample collected and sperm parameters. Supplementary Table S2: list of differentially methylated genes (n = 138) between score 6 and score 0 sperm samples [file 1434546.f1.zip › Supplementary Figure S1 (1).pdf]

**Figure S2**

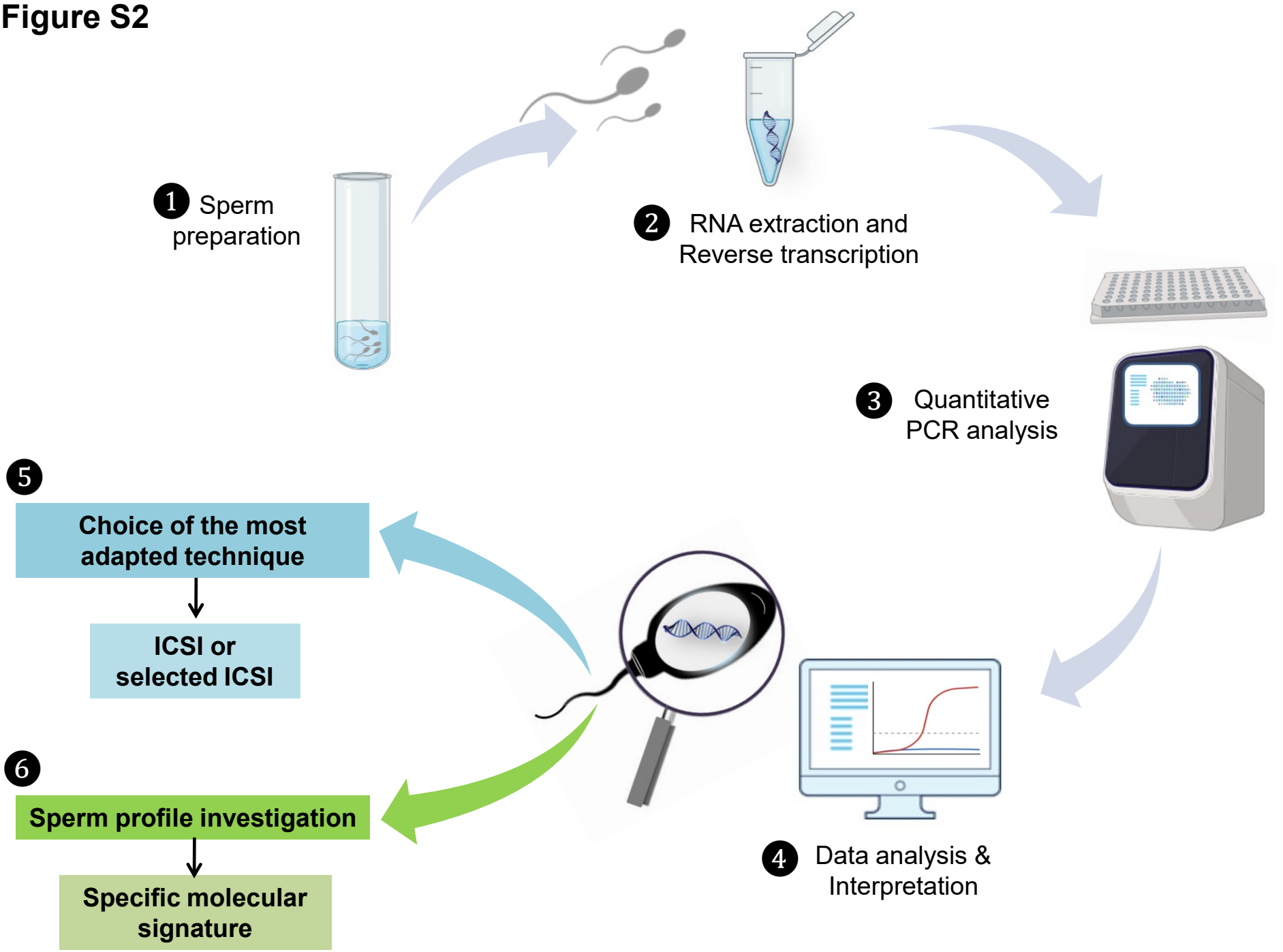

Supplement: Supplementary 1 — Supplementary Table S1 Sample collected and sperm parameters. Supplementary Table S2: list of differentially methylated genes (n = 138) between score 6 and score 0 sperm samples [file 1434546.f1.zip › Supplementary Figure S2 (1).pdf]
