# Supplementary material for: Molecular Profiling of Spermatozoa Reveals Correlations between Morphology and Gene Expression: A Novel Biomarker Panel for Male Infertility
Source: Biomed Res Int. 2021 Sep 18;2021:1434546. doi: 10.1155/2021/1434546 (PMC8485144; doi:10.1155/2021/1434546)
Supplement: Supplementary 2 — Supplementary Figure S1: expression values (in Log2 (RPKM)) of CCDC88B, CACNA1H, and CACNA1C in 30 tissues from the Genotype-Tissue Expression (GTEx) consortium. Supplementary Figure S2: envisioned sperm test for male infertility in ART. [file 1434546.f2.zip › Supplementary Table S1.pdf]

Table S1: Sample collections and sperm parameters

Samples	Age	Concentration (Million/ml)	Mobility (%)	Morphology (%)	Smoking	Scoring	Approches
P1	36	58 000 000	50	7	No	**Score 6**	**DNA Sequencing to identify candidate genes**
P2	57	65 000 000	45	6	No		
P3	30	90 000 000	45	2	No		
P4	39	16 000 000	10	1	No	**Score 0**	
P5	47	41 000 000	30	1	Yes		
P6	32	10 000 000	40	1	No		
P7	40	97 000 000	50	13	No	**Score 6**	**RT-qPCR**
P8	36	63 000 000	50	9	No		
P9	38	108 000 000	50	14	No		
P10	38	7 000 000	30	9	No		
P11	41	24 000 000	30	17	No		
P12	35	83 000 000	30	3	No	**Score 0**	
P13	45	2 000 000	5	1	No		
P14	32	55 000 000	40	1	No		
P15	29	28 000	40	5	No		
P16	49	65 000 000	50	3	Yes		
