# Supplementary material for: Molecular Profiling of Spermatozoa Reveals Correlations between Morphology and Gene Expression: A Novel Biomarker Panel for Male Infertility
Source: Biomed Res Int. 2021 Sep 18;2021:1434546. doi: 10.1155/2021/1434546 (PMC8485144; doi:10.1155/2021/1434546)
Supplement: Supplementary 2 — Supplementary Figure S1: expression values (in Log2 (RPKM)) of CCDC88B, CACNA1H, and CACNA1C in 30 tissues from the Genotype-Tissue Expression (GTEx) consortium. Supplementary Figure S2: envisioned sperm test for male infertility in ART. [file 1434546.f2.zip › Supplementary Table S2.pdf]

Table S2: List of differentially methylated genes (n=138) between score 6 and score 0 sperm sample

Chr.	start	end	strand	pvalue	meth.diff (%)	feature.id	Symbol
chr8	18432001	18432001	-	1,15E-23	65	NM_206909	PSD3
chr2	168675188	168675188	-	2,20E-23	59	NR_131227	LOC10561
chr1	180138799	180138799	+	3,79E-20	61	NM_002826	QSOX1
chr9	84204401	84204401	-	7,14E-19	75	NM_00130310	TLE1
chr4	2401549	2401549	-	1,06E-17	75	NM_00117265	ZFYVE28
chr11	16424656	16424656	-	2,65E-17	38	NM_00114581	SOX6
chr4	52940625	52940625	+	4,90E-17	60	NM_00129760	SPATA18
chr2	233734082	233734082	+	5,26E-16	62	NM_00134612	SNORC
chr13	111280711	111280711	+	1,16E-14	51	NM_00124288	NAXD
chr1	246132032	246132032	-	1,24E-14	43	NM_022743	SMYD3
chr10	65276946	65276946	-	4,72E-14	46	NM_00131815	JMJD1C
chr1	156624237	156624237	+	5,63E-14	70	NM_021948	BCAN
chr8	67348906	67348906	+	1,05E-13	38	NM_144650	ADHFE1
chr13	94822635	94822635	+	3,40E-13	38	NM_005708	GPC6
chr18	44333133	44333133	-	3,72E-13	50	NM_013305	ST8SIA5
chr8	144437295	144437295	-	3,76E-13	46	NM_00125844	TOP1MT
chr6	46110404	46110404	+	9,50E-13	75	NM_014936	ENPP4
chr6	152547152	152547152	-	1,88E-12	51	NM_182961	SYNE1
chr2	204107024	204107024	+	3,26E-12	65	NM_177538	CYP20A1
chr11	68699774	68699774	+	4,78E-12	50	NM_002180	IGHMBP2
chr16	1507779	1507779	-	5,59E-12	71	NM_00111433	CLCN7
chr6	604607	604607	-	1,13E-11	32	NR_073064	EXOC2
chr13	101322623	101322623	-	1,22E-11	57	NM_032813	TMTC4
chr1	234370652	234370652	+	1,40E-11	29	NM_00130084	SLC35F3
chr8	63516586	63516586	+	1,94E-11	45	NM_00130453	NKAIN3
chr7	36320213	36320213	+	4,93E-11	55	NM_030636	EEPD1
chr16	2025601	2025601	+	5,22E-11	58	NM_006453	TBL3
chr8	130366060	130366060	-	6,44E-11	66	NR_130919	CCDC26
chr14	47872289	47872289	-	7,45E-11	37	NM_00111349	MDGA2
chr10	53627347	53627347	+	9,44E-11	39	NM_006258	PRKG1
chr12	44292746	44292746	+	1,08E-10	31	NM_032256	TMEM117
chr10	3148129	3148129	+	2,13E-10	58	NM_00134594	PFKP
chr3	111676660	111676660	+	2,74E-10	50	NM_00113443	PHLDB2
chr2	3688860	3688860	+	2,92E-10	34	NM_00125598	COLEC11
chr20	54944792	54944792	-	3,07E-10	65	NM_198433	AURKA
chr12	5775665	5775665	-	3,51E-10	54	NM_00127859	ANO2
chr4	7437058	7437058	+	3,60E-10	46	NM_020777	SORCS2
chr13	111107860	111107860	+	5,88E-10	45	NM_001846	COL4A2
chr1	34328551	34328551	+	8,85E-10	67	NM_145205	HMGB4
chr2	239012831	239012831	+	9,55E-10	55	NM_194312	ESPNL
chr7	1950810	1950810	-	9,71E-10	55	NM_00130452	MAD1L1
chr6	7907885	7907885	-	1,26E-09	33	NR_037616	BLOC1S5-
chr6	7907885	7907885	-	1,26E-09	33	NM_030810	TXNDC5
chr5	31798797	31798797	+	1,28E-09	30	NM_178140	PDZD2
chr18	72995536	72995536	+	1,33E-09	38	NM_00130821	TSHZ1
chr1	178751230	178751230	+	1,40E-09	58	NM_00128624	RALGPS2
chr7	76623243	76623243	+	2,05E-09	27	NR_023383	DTX2P1-U

chr6	162472372	162472372	-	2,41E-09	55	NM_004562	PRKN
chr6	159205633	159205633	-	2,83E-09	57	NM_00111107	EZR
chr3	57681083	57681083	+	3,64E-09	48	NR_135550	DENND6A-
chr16	87647505	87647505	+	4,08E-09	48	NM_020655	JPH3
chr6	41519653	41519653	+	5,47E-09	36	NM_00101242	FOXP4
chr2	10942688	10942688	-	5,89E-09	41	NM_00128270	PDIA6
chr7	138481804	138481804	-	6,41E-09	54	NM_020632	ATP6V0A4
chr6	5135884	5135884	+	6,64E-09	42	NR_126015	LYRM4-AS
chr2	71361179	71361179	+	7,48E-09	34	NM_005791	MPHOSPH
chr5	359545	359545	+	7,70E-09	36	NM_020731	AHRR
chr8	134116852	134116852	+	7,83E-09	33	NM_003235	TG
chr11	64112393	64112393	+	9,51E-09	38	NM_032251	CCDC88B
chr12	2451161	2451161	+	1,04E-08	26	NM_199460	CACNA1C
chr3	187421035	187421035	+	1,18E-08	36	NR_034062	LOC10013
chr6	161675951	161675951	-	1,19E-08	64	NM_020133	AGPAT4
chr7	70172411	70172411	+	1,19E-08	58	NM_00112723	AUTS2
chr3	43026742	43026742	+	1,53E-08	41	NM_00112990	FAM198A
chr2	240241122	240241122	-	1,86E-08	49	NM_006037	HDAC4
chr11	118528465	118528465	+	1,99E-08	38	NM_00114475	PHLDB1
chr4	154174325	154174325	+	2,10E-08	33	NM_015271	TRIM2
chr2	1233640	1233640	+	3,32E-08	26	NM_018968	SNTG2
chr5	238008	238008	+	3,47E-08	42	NM_004168	SDHA
chr1	9382803	9382803	+	4,06E-08	52	NM_025106	SPSB1
chr6	116262857	116262857	-	4,42E-08	50	NM_002031	FRK
chr5	132574470	132574470	-	4,77E-08	45	NM_015082	FSTL4
chr12	1937877	1937877	+	5,06E-08	35	NM_00116392	LRTM2
chr10	128974622	128974622	-	5,10E-08	38	NM_00103976	FAM196A
chr6	168941700	168941700	+	5,94E-08	49	NM_00116641	SMOC2
chr3	124912462	124912462	-	6,33E-08	44	NM_024628	SLC12A8
chr12	119836063	119836063	+	7,68E-08	55	NM_178499	CCDC60
chr14	77741395	77741395	-	7,85E-08	35	NM_013382	POMT2
chr10	93371129	93371129	-	8,23E-08	36	NR_024467	HECTD2-A
chr14	75034700	75034700	-	8,46E-08	49	NM_000428	LTBP2
chr9	73532350	73532350	-	8,49E-08	43	NM_00100747	TRPM3
chr12	52078762	52078762	+	8,94E-08	41	NM_00133026	SCN8A
chr8	11578850	11578850	+	8,94E-08	33	NM_00130809	GATA4
chr19	2354910	2354910	+	9,88E-08	39	NM_152988	SPPL2B
chr10	134724843	134724843	-	1,05E-07	50	NM_00120004	CFAP46
chr8	1020811	1020811	+	1,25E-07	47	NM_00134681	DLGAP2
chr10	44064567	44064567	-	1,27E-07	57	NM_00132434	ZNF239
chr7	7678632	7678632	-	1,54E-07	40	NM_002947	RPA3
chr13	113713904	113713904	+	1,59E-07	44	NM_00132081	MCF2L
chr8	142200466	142200466	+	1,67E-07	39	NR_148197	DENND3
chr16	2084214	2084214	+	1,71E-07	34	NM_00113001	SLC9A3R2
chr1	171156996	171156996	+	1,74E-07	54	NM_00130134	FMO2
chr12	72670724	72670724	+	1,78E-07	37	NM_013381	TRHDE
chr12	69950545	69950545	+	2,10E-07	49	NM_00127835	FRS2
chr1	246810612	246810612	+	2,22E-07	53	NM_152609	CNST
chr7	143320985	143320985	+	2,31E-07	30	NM_173678	TCAF2
chr19	5455795	5455795	+	2,34E-07	49	NM_181710	ZNRF4
chr16	1255390	1255390	+	2,55E-07	55	NM_021098	CACNA1H

chr12	8386879	8386879	-	2,58E-07	28	NR_024254	FAM86FP
chr1	43412728	43412728	-	2,59E-07	50	NM_006516	SLC2A1
chr14	73355132	73355132	-	2,65E-07	41	NM_00128054	DPF3
chr14	104625669	104625669	+	2,71E-07	39	NM_015656	KIF26A
chr7	157595166	157595166	-	2,81E-07	32	NM_00130826	PTPRN2
chr1	57884310	57884310	-	2,85E-07	30	NM_00135398	DAB1
chr6	147570723	147570723	+	2,88E-07	49	NM_139244	STXBP5
chr11	2690532	2690532	-	3,08E-07	39	NR_002728	KCNQ1OT
chr17	46952510	46952510	-	3,11E-07	32	NR_135674	LOC10537
chr4	529908	529908	+	3,34E-07	51	NM_00128905	PIGG
chr6	13173146	13173146	+	4,40E-07	33	NM_00132231	PHACTR1
chr12	133417909	133417909	-	5,13E-07	45	NM_00116134	CHFR
chr5	172314090	172314090	+	5,37E-07	44	NM_00103171	ERGIC1
chr4	1186495	1186495	-	6,04E-07	32	NM_00119902	SPON2
chr3	120318272	120318272	+	6,25E-07	32	NM_00116833	NDUFB4
chr6	40387745	40387745	-	6,35E-07	31	NM_020737	LRFN2
chr7	101688628	101688628	+	6,42E-07	35	NM_181552	CUX1
chr1	21031043	21031043	-	6,46E-07	29	NM_00112281	KIF17
chr2	235953838	235953838	+	6,50E-07	50	NM_014521	SH3BP4
chr16	8949221	8949221	-	6,58E-07	30	NM_00127826	CARHSP1
chr3	119243934	119243934	-	7,48E-07	44	NM_005191	CD80
chr5	21488827	21488827	+	8,23E-07	29	NR_027028	GUSBP1
chr16	84776300	84776300	+	8,84E-07	45	NR_073578	USP10
chr10	133913276	133913276	+	9,31E-07	35	NM_00132308	JAKMIP3
chr17	71432883	71432883	-	9,72E-07	32	NM_00114495	SDK2
chr17	80877861	80877861	+	1,00E-06	28	NM_005993	TBCD
chr2	242946523	242946523	+	1,01E-06	56	NR_149023	LOC28509
chr8	11986406	11986406	+	1,11E-06	41	NR_027425	FAM66D
chr5	10376929	10376929	+	1,17E-06	27	NM_005885	MARCH6
chr8	129031341	129031341	+	1,18E-06	52	NR_003367	PVT1
chr17	7645325	7645325	+	1,28E-06	36	NM_020877	DNAH2
chr6	138655905	138655905	+	1,43E-06	33	NM_020340	ARFGEF3
chr5	1291880	1291880	-	1,48E-06	40	NR_149163	TERT
chr10	43746702	43746702	-	1,53E-06	49	NM_145313	RASGEF1
chr10	3710980	3710980	+	1,61E-06	46	NR_131187	LOC10537
chr12	1940350	1940350	-	1,64E-06	52	NM_172364	CACNA2D
chr4	170913057	170913057	-	1,68E-06	48	NM_00100955	MFAP3L
chr15	93609391	93609391	-	1,76E-06	33	NM_00116628	RGMA
chr17	743989	743989	-	1,79E-06	25	NM_022463	NXN
chr16	87988571	87988571	+	1,85E-06	33	NM_00117353	BANP

s.

Name	feature.ensembl	ture.exon_intron
pleckstrin and Sec7 domain containing 3		intron
uncharacterized LOC105616981		intron
quiescin sulfhydryl oxidase 1		intron
transducin like enhancer of split 1		intron
zinc finger FYVE-type containing 28		intron
SRY-box 6	ENSG00000110693	intron
spermatogenesis associated 18	ENSG00000163071	intron
upstream in-frame stop codon		intron
NAD(P)HX dehydratase		intron
SET and MYND domain containing 3		intron
jumonji domain containing 1C	ENSG00000171988	intron
brevican	ENSG00000132692	intron
alcohol dehydrogenase, iron containing 1		intron
glypican 6	ENSG00000183098	intron
upstream in-frame stop codon		intron
DNA topoisomerase I mitochondrial		intron
ectonucleotide pyrophosphatase/phosphodiesterase 4		intron
spectrin repeat containing nuclear envelope pro	ENSG00000131018	intron
cytochrome P450 family 20 subfamily A membe	ENSG00000119004	intron
immunoglobulin mu binding protein 2	ENSG00000132740	intron
chloride voltage-gated channel 7	ENSG00000103249	intron
exocyst complex component 2	ENSG00000112685	intron
transmembrane and tetratricopeptide repeat co	ENSG00000125247	exon (2/19)
solute carrier family 35 member F3		intron
sodium/potassium transporting ATPase interacting 3		intron
upstream in-frame stop codon		intron
transducin beta like 3		exon (10/22)
CCDC26 long non-coding RNA		intron
upstream in-frame stop codon		intron
protein kinase, cGMP-dependent, type I	ENSG00000185532	intron
transmembrane protein 117		intron
phosphofructokinase, platelet	ENSG00000067057	intron
pleckstrin homology like domain family B member 2		intron
collectin subfamily member 11	ENSG00000118004	intron
aurora kinase A	ENSG00000087586	exon (11/11)
anoctamin 2		intron
		intron
collagen type IV alpha 2 chain	ENSG00000134871	intron
high mobility group box 4		exon (2/2)
espin like		intron
mitotic arrest deficient 1 like 1	ENSG00000002822	intron
BLOC1S5-TXNDC5 readthrough (NMD candidate)		intron
thioredoxin domain containing 5		intron
PDZ domain containing 2	ENSG00000133401	intron
teashirt zinc finger homeobox 1	ENSG00000179981	intron
Ral GEF with PH domain and SH3 binding motif 2		intron
PK3BP1-PMS2P11		intron

parkin RBR E3 ubiquitin protein ligase	ENSG00000185345	intron
ezrin	ENSG00000092820	intron
DENND6A divergent transcript		exon (2/2)
junctophilin 3	ENSG00000154118	intron
forkhead box P4		intron
protein disulfide isomerase family A member 6		exon (3/14)
ATPase H+ transporting V0 subunit a4	ENSG00000105929	intron
LYRM4 antisense RNA 1		intron
M-phase phosphoprotein 10		exon (3/11)
aryl-hydrocarbon receptor repressor	ENSG00000063438	intron
thyroglobulin	ENSG00000042832	intron
coiled-coil domain containing 88B		exon (14/27)
calcium voltage-gated channel subunit alpha1 (	ENSG00000151067	intron
hCG1645011-like		intron
1-acylglycerol-3-phosphate O-acyltransferase 4		intron
AUTS2, activator of transcription and developm	ENSG00000158321	intron
family with sequence similarity 198 member A		intron
histone deacetylase 4	ENSG00000068024	intron
pleckstrin homology like domain family B member 1		exon (23/23)
tripartite motif containing 2	ENSG00000109654	intron
syntrophin gamma 2	ENSG00000172554	intron
succinate dehydrogenase complex flavoprotein	ENSG00000073578	intron
		intron
fyn related Src family tyrosine kinase		exon (8/8)
follistatin like 4		intron
leucine rich repeats and transmembrane domains 2		intron
family with sequence similarity 196 member A		exon (4/6)
SPARC related modular calcium binding 2	ENSG00000112562	intron
solute carrier family 12 member 8	ENSG00000221955	intron
coiled-coil domain containing 60		intron
protein O-mannosyltransferase 2	ENSG00000009830	exon (21/21)
HECTD2 antisense RNA 1		exon (1/5)
latent transforming growth factor beta binding p	ENSG00000119681	intron
transient receptor potential cation channel subf	ENSG00000083067	intron
sodium voltage-gated channel alpha subunit 8	ENSG00000196876	intron
GATA binding protein 4	ENSG00000136574	intron
signal peptide peptidase like 2B	ENSG00000005206	exon (16/16)
cilia and flagella associated protein 46		intron
DLG associated protein 2	ENSG00000198010	intron
zinc finger protein 239		intron
replication protein A3		intron
MCF.2 cell line derived transforming sequence like		intron
DENN domain containing 3		exon (21/24)
SLC9A3 regulator 2	ENSG00000065054	intron
flavin containing monooxygenase 2		intron
thyrotropin releasing hormone degrading enzyr	ENSG00000072657	intron
fibroblast growth factor receptor substrate 2		intron
consortin, connexin sorting protein		exon (9/11)
TRPM8 channel associated factor 2		intron
zinc and ring finger 4		exon (1/1)
calcium voltage-gated channel subunit alpha1 (	ENSG00000196557	intron

		exon (4/5)
solute carrier family 2 member 1	ENSG00000117394	intron
double PHD fingers 3		intron
kinesin family member 26A		intron
protein tyrosine phosphatase, receptor type N2	ENSG00000155093	intron
DAB1, reelin adaptor protein	ENSG00000173406	intron
syntaxin binding protein 5		intron
KCNQ1 opposite strand/antisense transcript 1	ENSG0000026982	exon (1/1)
uncharacterized LOC105371814		intron
phosphatidylinositol glycan anchor biosynthesis	ENSG00000174227	intron
phosphatase and actin regulator 1		intron
checkpoint with forkhead and ring finger domai	ENSG00000072609	exon (16/16)
		intron
spondin 2		intron
NADH:ubiquinone oxidoreductase subunit B4		intron
upstream in-frame stop codon		intron
cut like homeobox 1	ENSG00000257923	intron
kinesin family member 17		exon (5/15)
SH3 domain binding protein 4	ENSG00000130147	intron
calcium regulated heat stable protein 1		intron
CD80 molecule		exon (7/7)
glucuronidase, beta pseudogene 1		intron
ubiquitin specific peptidase 10		intron
Janus kinase and microtubule interacting protein 3		intron
sidekick cell adhesion molecule 2		intron
tubulin folding cofactor D	ENSG00000141556	intron
uncharacterized FLJ38379		exon (1/1)
family with sequence similarity 66 member D		intron
membrane associated ring-CH-type finger 6		intron
Pvt1 oncogene (non-protein coding)		intron
dynein axonemal heavy chain 2		intron
ARFGEF family member 3		exon (33/34)
telomerase reverse transcriptase	ENSG00000164362	intron
RasGEF domain family member 1A		intron
uncharacterized LOC105376360		intron
calcium voltage-gated channel auxiliary subuni	ENSG00000151062	intron
microfibril associated protein 3 like		exon (2/2)
repulsive guidance molecule BMP co-receptor a		intron
nucleoredoxin		intron
BTG3 associated nuclear protein	ENSG00000172530	intron
